# A glaucoma micro-stent with diverging channel and stepped shaft structure based on microfluidic template processing technology

**DOI:** 10.1186/s12938-024-01266-4

**Published:** 2024-07-27

**Authors:** Chen Wang, Fule Wang, Yunru Liao, Chengguo Zuo, Mingkai Lin, Kemin Wang, Dongni Ren, Hongbo Zhang, Ruixue Yin

**Affiliations:** 1 School of Mechanical and Power Engineering, East China University of Science and Technology, No. 130 Meilong Road, Shanghai, 200237 China; 2 State Key Laboratory of Ophthalmology, Zhongshan Ophthalmic Center, Sun Yat-Sen University, No.7 Jinsui Road, Tianhe District, Guangzhou, 510060 China; 3 Department of Ophthalmology, Sun Yat-Sen Memorial Hospital, Sun Yat-Sen University, Guangzhou, China; 4 Mingche Biotechnology Co., Ltd, Suzhou, 215000 China

**Keywords:** Glaucoma, MIGS, Drainage stent, Microfluidic channel processing, PDMS, Drainage efficiency

## Abstract

**Background:**

Minimally invasive glaucoma surgery (MIGS) has experienced a surge in popularity in recent years. Glaucoma micro-stents serve as the foundation for these minimally invasive procedures. Nevertheless, the utilization of these stents still presents certain short-term and long-term complications. This study aims to elucidate the creation of a novel drainage stent implant featuring a diverging channel, produced through microfluidic template processing technology. Additionally, an analysis of the mechanical properties, biocompatibility, and feasibility of implantation is conducted.

**Results:**

The stress concentration value of the proposed stent is significantly lower, approximately two to three times smaller, compared to the currently available commercial XEN gel stent. This indicates a stronger resistance to bending in theory. Theoretical calculations further reveal that the initial drainage efficiency of the gradient diverging drainage stent is approximately 5.76 times higher than that of XEN stents. Notably, in vivo experiments conducted at the third month demonstrate a favorable biocompatibility profile without any observed cytotoxicity. Additionally, the drainage stent exhibits excellent material stability in an in vitro simulation environment.

**Conclusions:**

In summary, the diverging drainage stent presents a novel approach to the cost-effective and efficient preparation process of minimally invasive glaucoma surgery (MIGS) devices, offering additional filtering treatment options for glaucoma.

## Background

Glaucoma, a neurodegenerative disease, is the second most common cause of blindness worldwide and the leading cause of irreversible blindness. According to statistics, the global prevalence of glaucoma was estimated to be 64.3 million in 2013, with projections of 76.0 million in 2020 and 111.8 million in 2040 for individuals aged 40–80 years [1]. The pathogenesis of glaucoma involves the buildup of aqueous humor in the anterior chamber, resulting in elevated intraocular pressure (IOP) and subsequent permanent damage to optic nerve cells. Typical characteristics of glaucoma include optic papilla injury and associated visual field defects [2, 3]. The treatment of glaucoma has always focused on reducing intraocular pressure, minimizing optic nerve damage, and preserving visual function. Laser trabeculoplasty may be utilized when traditional drug therapy is ineffective or patients cannot tolerate long-term medication. Minimally invasive glaucoma surgery (MIGS) should be considered if laser therapy fails to achieve a safe reduction in intraocular pressure or when maximally tolerated medical therapy fails. Trabeculectomy, a more invasive procedure, is considered as a last resort when none of the aforementioned treatments yield satisfactory results[4–6].

Minimally invasive glaucoma surgery (MIGS) was developed to address the increasing need for long-term management of this chronic disease [7, 8]. MIGS aims to minimize damage to the conjunctiva and sclera, enhance aqueous humor outflow through various techniques, and ultimately reduce intraocular pressure (IOP). The advantages of MIGS devices are significant and can be attributed to the following factors: 1. lowered intraocular pressure and reduced complications compared to traditional glaucoma surgery; 2. improved treatment outcomes leading to decreased postoperative pain and discomfort for patients; 3. shorter learning curve for surgeons compared to traditional surgery; 4. expanded indications, allowing for combined glaucoma cataract surgery with positive effects on refractory and complex glaucoma cases; 5. compatibility with other traditional glaucoma surgeries (e.g., trabeculectomy [9, 10] or glaucoma drainage implantation [11]) even after MIGS.

Polymer hydrogel materials, known for their flexibility, excellent biocompatibility, and minimal foreign body sensation after implantation, have become the primary materials used in MIGS glaucoma drainage stents. Examples include the XEN Gel Stent [12, 13] and PRESERFLO [14, 15]. However, the production of polymer devices primarily relies on molding techniques like spin-coating or extrusion, which involve intricate and stringent processes, presenting considerable challenges to batch stability. Furthermore, due to the small size of the device and the limitations of the single molding method, the shape and inner diameter of the drainage stent cannot be easily customized, resulting in the potential for low intraocular pressure (IOP) with traditional equal-diameter stents [16–18].

In this study, we designed and fabricated a stepped drainage stent with gradually expanding inner diameter. It aims to address the challenges of high surgical invasiveness, complexity in manufacturing existing MIGS drainage devices, and various complications post-implantation. The outcomes achieved include higher aqueous humor drainage efficiency, prevention of complications such as aqueous humor reflux and low intraocular pressure, and reduced likelihood of device displacement within the eye. The device is manufactured using microfluidic templating technology and assembled with a customized injection system for integrated use. We carried out a 12-month in vitro degradation study to evaluate the long-term stability of the stent, systematically evaluated the mechanical properties and flow characteristics of the device by finite element analysis and fluid theory calculations. Subsequently, biocompatibility assessments were conducted, including in vitro cytotoxicity testing and histological examination three months after implantation of the animal model. Lastly, the device was implanted in the right eye of four rabbits, and their progress was meticulously monitored and examined for preliminary evidence supporting the surgical feasibility and short-term safety post-implantation.

## Results

### Characterization of the stents

**Fig. 1:**
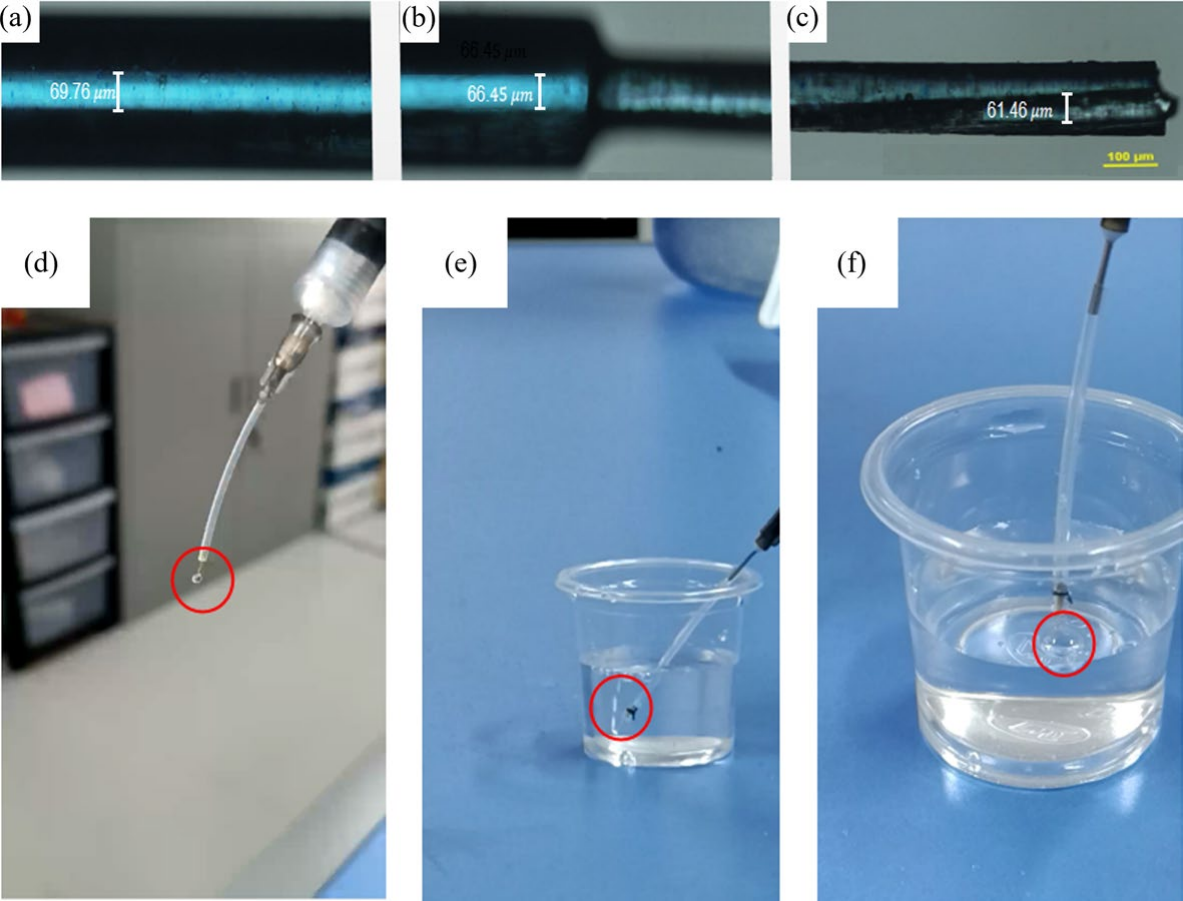
Microscopic view of **a** the anterior, **b** transitional, and **c** caudal segments of the drainage; Experimental setup for drainage stent conduction. **d** The syringe is slowly pushed, causing the stent to overflow at the outlet. **e, f** The syringe is slowly pushed, causing the stent to bubble up in pure water

After preparing the drainage stent, it underwent surface treatment, drying, and sterilization. The peripheral size of the stent was measured and shown in Fig. 1, which provides a microscopic view of its anterior (a), transitional (b), and caudal (c) segments. The design specifications of the stent are 6 mm in total length and the outer diameter is stepped in a pattern. The end with a larger outer diameter has an inner diameter of 60 μm, and the other end with a smaller outer diameter has an inner diameter of 70 μm. The actual dimensions of the stent were characterized by microscope. The maximum inner diameter is 69.76 μm, the minimum inner diameter is 61.46 μm, and at a certain section in the middle segment, the inner diameter is 66.45 μm. The actual diameter of the cylindrical inner lumen closely matches the design specifications. Additionally, a drainage stent conduction experiment was conducted as shown in Fig. 1d–f. The experimental results demonstrated that when the syringe was slowly pushed, the stent smoothly overflowed at the outlet (d), and in pure water, it generated uniform bubbles (e) and (f), indicating excellent conductivity of the stent.

### *In vitro degradation of the stents*

**Fig. 2:**
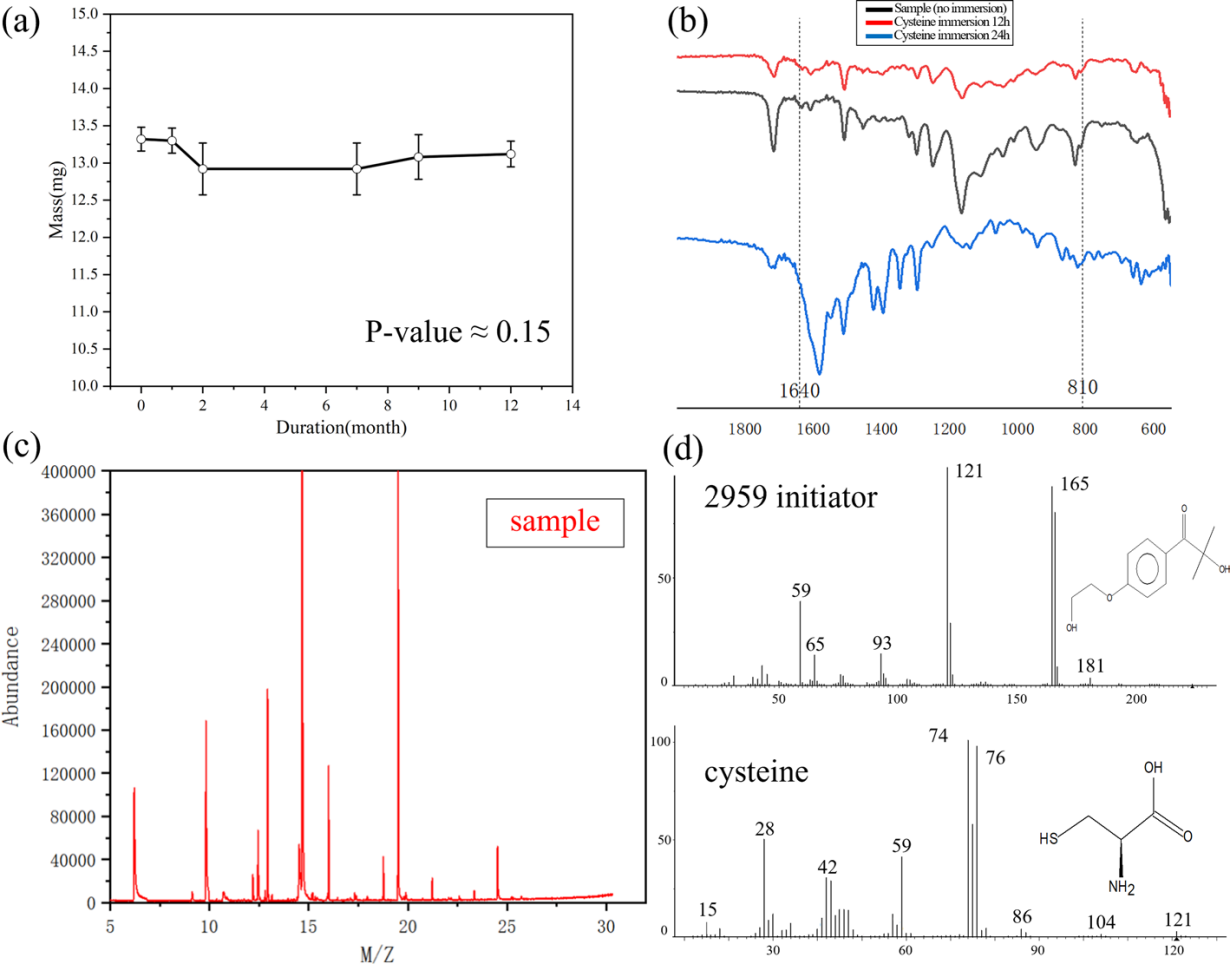
**a** Degradation results of the stent over a period of 12 months (*p*-value > 0.05, indicating that there was no significant difference). **b** IR spectra showing changes of double bonds on the surface of the stent after immersion in cysteine. **c** Gas chromatography–mass spectra of the sample extract. **d** Mass spectra of 2959 initiator and cysteine in the standard library

The stability of drainage stent implantation was investigated by analyzing the degradation rate and precipitate of the stents using weighing and gas chromatography–mass spectrometry. A one-year follow-up study of the drainage stents was conducted, and the results are presented in Fig. 2a. The average degradation rate of the drainage stent over 12 months was calculated to be 1.5%. At the same time, the analysis of the degradation data showed that the *p*-value > 0.05, indicating that there was no significant difference in the data, and it also indicated that the material of the stent had excellent resistance to degradation.

During the manufacturing process, the drainage stents underwent cysteine-soaked surface treatment and the introduction of photosensitizer. The residue of harmful substances on the stent after surface treatment was further evaluated by extracting the stent extract for infrared spectroscopy measurement and directly analyzing the stent precipitate using gas chromatography–mass spectrometry. The infrared spectrum (Fig. 2b) showed that the carbon–carbon double bonds at peaks 810 cm^−1^ and 1640 cm^−1^ disappeared after cysteine treatment, indicating the absence of viable double bonds on the surface of the drainage stent. The mass spectrum of the sample (Fig. 2c) shows a range of ion abundance peaks corresponding to mass-to-charge ratios (m/z). To determine the presence of residual 2959 initiator and cysteine in the sample, it is necessary to compare whether the ion peaks on the sample mass spectrum match the ion peaks on the 2959 initiator and cysteine standard mass spectra (Fig. 2d). In the mass spectrum of 2959 initiator, there are several characteristic peaks, such as the peak of m/z = 121, which appears to be the most significant. In the mass spectrum of cysteine, we see a high intensity of m/z = 74, which may be a landmark peak. There were no matching peaks in the mass spectrum of the sample, confirming the absence of 2959 initiator and cysteine in the stent extract.

### Flow characteristics within the stents

The drainage efficiency of the equal-diameter drainage stent and the diverging drainage stent can be visually compared by comparing their initial discharge rates. The equal-diameter drainage stent has an inner diameter of 65 μm, while the diverging drainage stent has a large end diameter of 70 μm and a small end diameter of 60 μm. The resulting equal-diameter drainage stent had an initial discharge rate of 17.75 μl/min, while the diverging drainage stent had a rate of 23.52 μl/min. The diverging drainage stent has a drainage efficiency that is approximately 32% higher than that of the equal-diameter stent. The commercial stent XEN has an inner diameter of 45 μm, resulting in an initial discharge rate of 4.08 μl/min. The initial drainage efficiency of the diverging drainage stent is 5.76 times higher than that of XEN stents. This is due to the fact that the gradually expanding cross-section can reduce the flow rate of the liquid in the pipeline, reduce the frictional resistance and resistance loss in the pipeline, and make it difficult for the liquid to accumulate during the flow process, thus improving the drainage efficiency. Additionally, calculations have shown that a 60–70 μm internal diameter stent provides 2–3 mmHg of resistance against aqueous humor production rates of 2–2.5 μl/min. Moreover, the episcleral venous pressure contributes 8 mmHg of pressure, so the fluid reflux needs to overcome a large resistance, which effectively prevents the occurrence of low intraocular pressure.

### Mechanical properties of the stents

COMSOL Multiphysics 5.6 was used to analyze the stress concentrations that occur when a draining stent is bent and deformed after implantation due to the presence of corneal curvature. Assuming no offset occurs after implanting the drainage stent, we set the end face of the stent inlet shaft as a fixed support and apply a radial force to the second half. Assuming no offset occurs after implanting the drainage stent, we set the end face of the stent inlet shaft as a fixed support and apply a radial force to the second half.

**Fig. 3:**
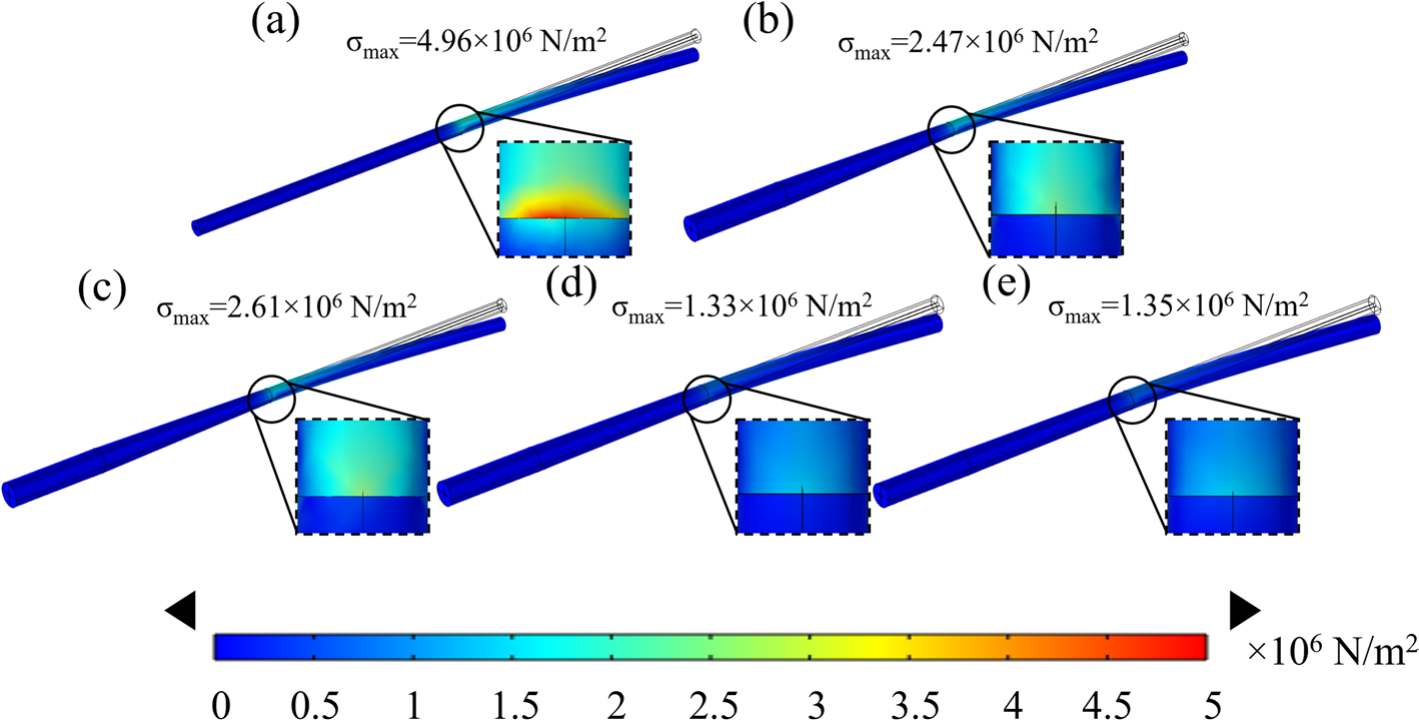
The mechanical simulation results. **a** The XEN gel stent; **b** the outer diameter varies along the shaft: 250 μm over 1 mm, reducing to 150 μm over 3 mm, bridged by a 2 mm transitional segment. The inner diameter fluctuates non-uniformly between 60 and 70 μm. **c** The outer diameter varies along the shaft: 250 μm over 1 mm, reducing to 150 μm over 3 mm, bridged by a 2 mm transitional segment, equal inner diameter 65 μm. **d** The outer diameter varies along the shaft: 250 μm over 1 mm, reducing to 200 μm over 3 mm, bridged by a 2 mm transitional segment. The inner diameter fluctuates non-uniformly between 60 and 70 μm. **e** The outer diameter varies along the shaft: 250 μm over 1 mm, reducing to 200 μm over 3 mm, bridged by a 2 mm transitional segment, equal inner diameter 65 μm

Figure 3 presents the mechanical simulation results for the XEN stent and the diverging drainage stent proposed in this work. Comparing Fig. 3b to Fig. 3c or Fig. 3d to Fig. 3e, for the stents with the same outer structure, the gradient inner diameter stent has a smaller maximum stress concentration than the equivalent inner diameter stent. Additionally, from the comparison of Fig. 3b, c with Fig. 3d, e, it can be seen that with the same external force, stents with smaller diameters show higher stress concentrations. Notably, the stress concentration of the proposed stents in Fig. 3b–e is 2–3 times lower than that of the popular commercial XEN stent in Fig. 3a, suggesting superior bending resistance theoretically.

### Biocompatibility of the stents

#### In vitro cytotoxicity

**Fig. 4:**
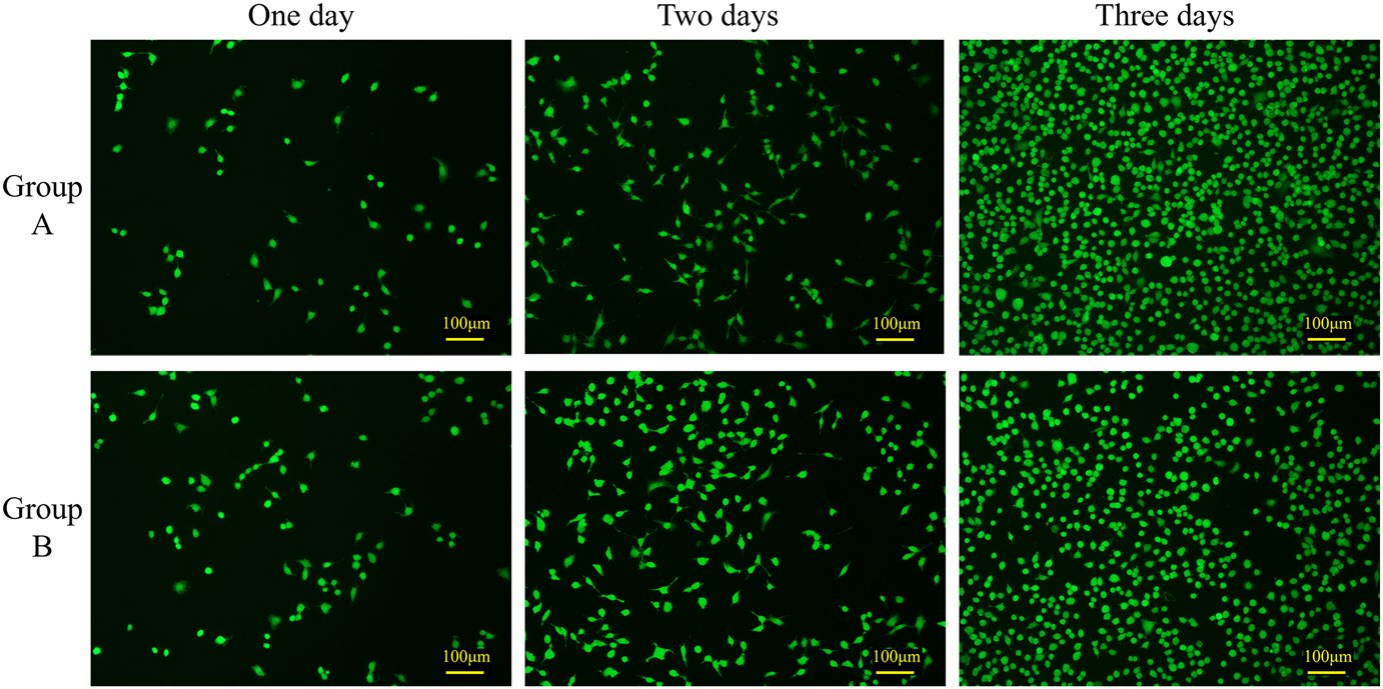
HUVEC cell culture subjected to dead and alive staining. Group A is the blank control group, and Group B is the experimental group treated with the extraction

Figure 4 illustrates the status of human umbilical vein endothelial cells (HUVEC) after three days of culture, showing the presence or absence of cell viability. The figure indicates that both the blank group and the experimental group exhibited healthy cell morphology and displayed a stable growth trend over the 3-day period. There were minimal observations of cell death within the field of view. These findings suggest that the drainage stent does not exhibit cellular toxicity.

#### Subcutaneous implantation experiments

**Fig. 5:**
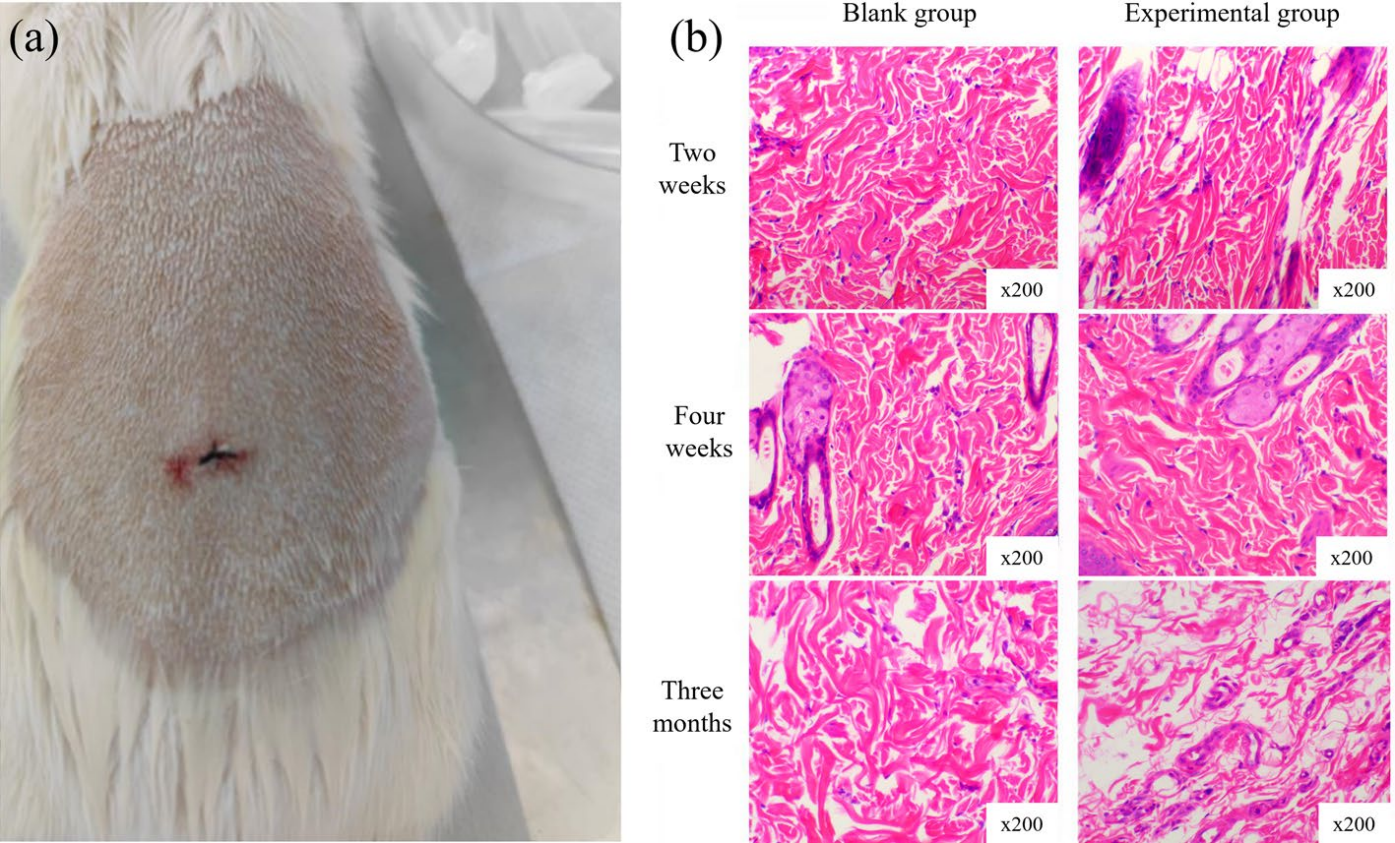
Subcutaneous implantation experiment. **a** SD rats were implanted with a drainage stent subcutaneously on the back; **b** histological section of the rat at the site of the implanted stent

In the study, an incision was made on the back of Sprague–Dawley (SD) rats, as depicted in Fig. 5. The rat subcutaneous implantation experiment was carried out by Shanghai Xiqi Biotechnology Center. A drainage stent was then implanted into the subcutaneous tissue. Tissue inflammation was observed by conducting sections at the second week, fourth week, and third month after the implantation. No inflammatory response was observed in the subcutaneous tissue at 3 months after implantation by comparison with the control group. This suggests that the drainage stent did not induce an inflammatory reaction in the long term.

### Stents performance in rabbit eyes in vivo

**Fig. 6:**
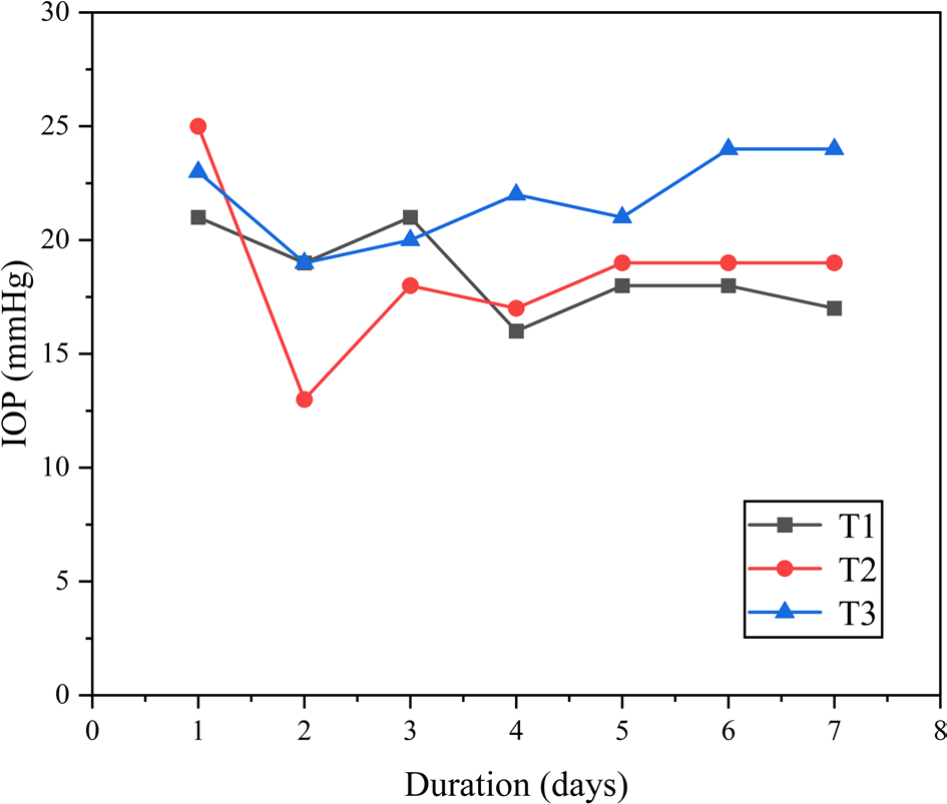
Fluctuations in intraocular pressure during the initial week following surgery

Based on the outcomes of animal experiments (Table 1), it was observed that the size of the filter vesicle remained relatively stable within one-week post-stent implantation, suggesting the stent’s efficacy in maintaining a consistent drainage function. Initially, there was a rapid decline in intraocular pressure post-implantation, followed by stabilization (Fig. 6). In addition, an increase in vascular density was observed after implantation. No stent displacement or complications such as low intraocular pressure or inflammation were reported. The animal experiment demonstrated the stent’s surgical feasibility and short-term implantation safety, paving the way for further trials [28, 29].

**Table 1:** Intraocular pressure, follicle size, and vascular density recorded for one-week post-stent implantation in rabbit eye

Duration	Groups			
	T1	T2	T3	T4
1 days (IOP/ filter bleb size/ vascular density)	21/8 × 5 × 2/2	25/8 × 5 × 3/2	23/8 × 5 × 2/2	Unchanged
2 days (IOP/ filter bleb size/ vascular density)	19/8 × 5 × 2/2	13/8 × 5 × 1/3	19/8 × 5 × 2/3	Unchanged
3 Days (IOP/ filter bleb size/ vascular density)	21/8 × 5 × 2/2	18/8 × 5 × 1/3	20/8 × 5 × 2/3	Unchanged
4 days (IOP/ filter bleb size/ vascular density)	16/8 × 5 × 2/2	17/8 × 5 × 1/3	22/8 × 5 × 2/3	Unchanged
5 days (IOP/ filter bleb size/ vascular density)	18/8 × 5 × 2/2	19/7 × 4 × 1/3	21/8 × 5 × 2/3	Unchanged
6 days (IOP/ filter bleb size/ vascular density)	18/8 × 5 × 2/2	19/7 × 4 × 1/3	24/8 × 5 × 2/3	Unchanged
7 days (IOP/ filter bleb size/ vascular density)	17/8 × 5 × 2/2	19/7 × 4 × 1/3	24/8 × 5 × 2/3	Unchanged

Unit IOP: mmHg; filter bleb size: mm × mm × mm; vascular density: grades 1–4 (lager numbers represent higher vessel density)

## Discussion

In conventional glaucoma filtration drainage procedures such as trabeculectomy and minimally invasive stenting, there is a risk of low intraocular pressure and associated complications, particularly within the first month after surgery. Severe visual loss can occur in up to 20 percent of patients [19]. Various approaches been explored to prevent postoperative low intraocular pressure, including surgical improvements and the use of check valves [20, 21]. However, these procedures are often complex, lack reproducibility, and do not completely eliminate the occurrence of low intraocular pressure. To address this issue, we designed a diverging drainage stent based on theoretical calculations. This unique design combines the resistance provided by the stent and the episcleral venous pressure, effectively preventing the occurrence of low intraocular pressure. The purpose of this design is to provide a more reliable and reproducible solution that mitigates the risk of postoperative complications associated with low intraocular pressure.

The human eyeball undergoes various movements on a daily basis, such as blinking, rubbing, accidental collisions, and rapid eye movement during sleep. These movements subject the eye tissues to complex stresses, which can potentially lead to displacement of intraocular implanted devices or cause trauma to the surrounding tissues. As a result, stringent requirements exist for the axial fixation of drainage stents. As shown in Figs. 1a–c and 8, we designed a stepped structure for the outer diameter of the stent, aiming to prevent in vivo dislocation. Conical contact provides a larger contact area compared to horizontal contact at the same horizontal length. When a stent is subjected to external forces, it experiences both the horizontal component of tissue and frictional forces. The combined effect of the horizontal component force and frictional force impedes the movement of the drainage stent. In contrast to commercial drainage stents, which are limited by processing technology and can only manufacture equal-diameter stents, the stepped structure for the outer diameter of the stent in this study may help reduce the displacement rate.

The choice of molding method is closely related to the properties of the selected material. The flexibility of the material is inversely proportional to its dimensional accuracy and processing difficulty. Materials with better biocompatibility often require higher processing requirements and have higher processing difficulties. Traditional machining methods used for metal materials are not suitable for materials with high flexibility and biocompatibility. Common processing methods for biocompatible materials, such as spin-coating, may not provide the desired flexibility in shaping the appearance of the molded material and may not strictly control the dimensional accuracy. In our study, we utilized microfluidic channel template processing technology to prepare the drainage stent. This method proved to be efficient, safe, and provided good molding accuracy. It also showed potential for mass production of the stents.

The chip packaging and infusion molding process involves a series of steps, as shown in FlowChart 8–12 processes (Fig. 9). It is important to note that the metal used for constructing the prototype cavity needs to be placed coaxially. The packaging process requires operating under a microscope, and since plasma treatment has a limited duration, it is crucial to quickly and accurately align the flow channels under the microscope. This ensures that the plasma effect does not weaken and maintains its sealing effect on the package. During the packaging process, special attention should be given to aligning the holes of the upper and lower molds. This alignment greatly impacts the appearance of the prototype. Additionally, the placement position of the metal used for constructing the prototype cavity directly affects the position of the prototype cavity. Therefore, it is essential to ensure accurate placement of the metal to achieve the desired results.

Several key considerations exist in the mold design stage. Firstly, it is crucial to accurately control the dimensional accuracy of the fabricated stent to ensure the functionality of the drainage stent. Additionally, the convenience of operation in the chip preparation process needs to be considered, such as easy mold removal and chip cutting. Furthermore, the small diameter of the inner diameter steel needle is a critical factor that directly affects the drainage efficiency of the stent. The needle must have a certain level of flexibility to prevent breakage during the process of placing it in the guide groove. During the packaging and molding stage, proficiency in packaging operations is essential. Plasma surface treatment is a delicate process. Skilled and fast operation is required to bond the upper and lower chip within the validity period of the plasma treatment. Maintaining the coaxiality of the steel needle is another challenge. To address this, a guiding bulge equal to or slightly larger than the diameter of the steel needle is provided in the chip mold. This design ensures the coaxiality of tungsten steel needle and stent.

The degradation properties of intraocular implants are crucial in minimally invasive glaucoma, as they directly impact the duration and biocompatibility of the implant [22]. The data presented in Fig. 1 demonstrate that the drainage stents exhibit excellent degradation properties, which are advantageous for long-term implantation. The precipitation test results indicate that the surface of the drainage stent no longer had viable double bonds surface treatment, which is beneficial for the long-term presence of the stent in the eye.

## Conclusion

Our primary objective is to develop a novel glaucoma drainage stent. The stress concentration value of this stent is theoretically 2–3 times smaller than that of the current commercially available XEN gel stent, indicating stronger bending resistance. The initial drainage efficiency of the diverging drainage stent is calculated to be 5.76 times higher than that of XEN stents, and the gradient diverging design effectively reduces stent displacement rates. Extensive testing has demonstrated good in vivo biocompatibility without any signs of cytotoxicity. The drainage stent also exhibits excellent material stability in simulated in vitro environments. The feasibility of the surgical procedure has been established, effectively stabilizing intraocular pressure while preventing low intraocular pressure occurrences. In conclusion, based on comprehensive testing data, the diverging drainage stent represents a new approach to low-cost and efficient preparation processes for minimally invasive glaucoma surgery (MIGS) devices. It also provides additional options for glaucoma filtering treatment. Future experiments will involve stent implantation by injection, aiming to simplify the procedure further and reduce surgical risks.

## Materials and methods

### Principle of design

The design process of a glaucoma drainage stent must address two main issues: fixation and safety within the eye, and drainage efficiency and stability. For intraocular fixation, it is essential that the drainage stent remains in place without movement, and its external shape should not cause any damage to the eye tissue. In this design, a gradient circular section tubular structure is adopted, as circular shapes have the smallest shape factor among different cross-sectional shapes. The smooth transition of the rounded outer surface significantly reduces tissue irritation and minimizes friction-induced bleeding that may occur due to human activity. And this stepped outer diameter design provides a larger contact area. When the stent is subjected to external force, it will bear the horizontal component of the tissue and the frictional force at the same time, which hinders the movement of the stent and effectively reduces the displacement rate of the stent. Drainage efficiency and stability are also critical considerations. Drainage efficiency refers to the ability of the stent to effectively drain aqueous humor per unit of time. To achieve this, numerical calculations of fluid parameters for low Reynolds number microfluidic flow within the stent are involved. In this study, we evaluate the drainage efficiency of the stent by calculating the outlet flow *Q* (calculated by Eq. 6). The inlet environment for all stents is consistently the anterior chamber, and a greater outlet flow indicates higher drainage efficiency of the stent. Comparing to a pure cylindrical lumen, the gradually expanded cross-section designed in this study offers enhanced efficiency in aqueous humor drainage while mitigating complications such as aqueous humor reflux and low intraocular pressure. When analyzing the flow characteristics within the stent, we assumed that the extra scleral venous pressure (EVP) in glaucoma patients would be 1000 Pa and the anterior chamber intraocular pressure would be 30 mmHg, which is approximately equal to 4000 Pa. Rate of aqueous humor production is 2–2.5 µl/min [13, 30].

**Fig. 7:**
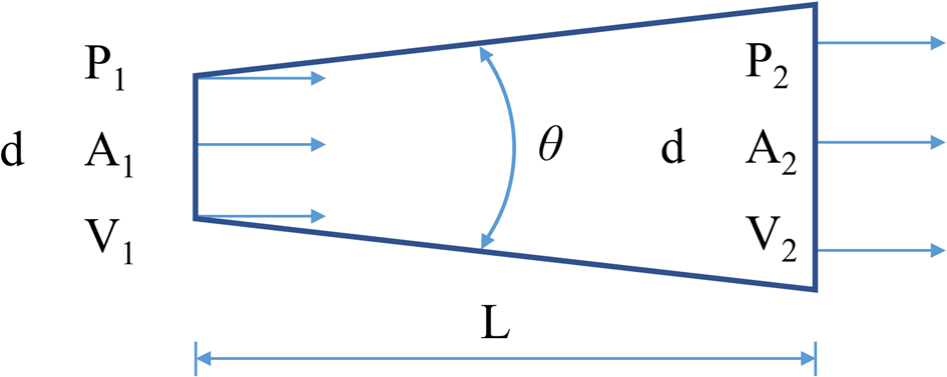
Section schematic diagram of fluid flowing through the pipeline

The dimensions of the drainage stent are determined by the principles of fluid mechanics. Figure 7 illustrates the fluid flow inside the tube. According to the principle of energy conservation, the fluid flow in the tube obeys Bernoulli's principle, which can be expressed as:

1 $${Z}_{1}+\frac{{P}_{1}}{\rho g}+\frac{{{V}_{1}}^{2}}{2g}={Z}_{2}+\frac{{P}_{2}}{\rho g}+\frac{{{V}_{2}}^{2}}{2g}+{H}_{L},$$

where *P*, *V*, ρ, *g* and *H*_*L*_ are the pressure, velocity of flow, aqueous humor density, acceleration due to gravity and loss of mechanical energy, respectively.

To traverse the anterior chamber and subconjunctival space, drainage stents typically need to have a length of approximately 6 to 8 mm. Due to the low rate of aqueous humor production, the tube’s diameter is typically kept below 100 μm to prevent low intraocular pressure. In the case of such a significant length–diameter ratio, the local resistance of the gradient pipe contributes less to the overall flow resistance, with distance friction becoming the primary factor. The loss of resistance can be described using Darcy's law:

2 $${H}_{L}=\xi \frac{{V}^{2}}{2g},$$

where ξ is the flow damping coefficient. The change in potential energy during horizontal flow can be ignored, and then Eq. 1 can be simplified as follows:

3 $$\frac{{P}_{1}}{\rho g}+\frac{{{V}_{1}}^{2}}{2g}=\frac{{P}_{2}}{\rho g}+\frac{{{V}_{2}}^{2}}{2g}+\xi \frac{{{V}_{2}}^{2}}{2g}.$$

It can be seen from the continuity equation:

4 $${V}_{1}={V}_{2}{\left(\frac{D}{d}\right)}^{2},\Delta P={P}_{1}-{P}_{2}={P}_{1}.$$

The outlet flow rate can then be derived as:

5 $${V}_{2}={\left(\frac{2{P}_{1}}{\rho }\right)}^\frac{1}{2}{\left[1+\xi -{\left(\frac{D}{d}\right)}^{4}\right]}^{-\frac{1}{2}}.$$

At this point, the outlet flow *Q* can be obtained, and the diffuser flow equation can be written as:

6 $$Q={V}_{2}{A}_{2}=\frac{\pi {D}^{2}}{4}{\left(\frac{2{P}_{1}}{\rho }\right)}^\frac{1}{2}{\left[1+\xi -{\left(\frac{D}{d}\right)}^{4}\right]}^{-\frac{1}{2}}.$$

Experimental evidence suggests that in microfluidics, the Reynolds number (Re) is typically small at small scales. When Re falls within the range of 1 < Re < (30–50), the drag coefficient of the diverging pipe can be calculated using the sudden expansion formula [23]:

7 $$\xi =\Delta P/\frac{\rho {{V}_{1}}^{2}}{2}=\frac{A}{\text{Re}},$$

where *A* is a function of diffusion angle and diffusion. When 2α is less than or equal to 40°, A can be expressed as:

8 $$A=\frac{20{n}_{1}^{0.33}}{{\left(tg\alpha \right)}^{0.75}},$$

where *n*_1_ is the entrance and exit area ratio. From the formulas above, the flow state of the drainage stent can be obtained in the initial implantation state.

Another important consideration in the design of drainage stents is selecting an appropriate inner diameter to ensure adequate flow resistance and prevent low intraocular pressure. The Hagen–Poiseuille equation can be used to calculate the flow resistance of the cylindrical tube, serving as the foundation for the design process:

9 $$\Delta P=\frac{8\upmu LQ}{\pi {R}^{4}},$$

where ∆*P*, μ, *L*, *Q* and *R* are the pressure loss along the lumen, dynamic viscosity, tube length, volume flow and tube radius, respectively.

### Flexural strength of the stent

**Fig. 8:**
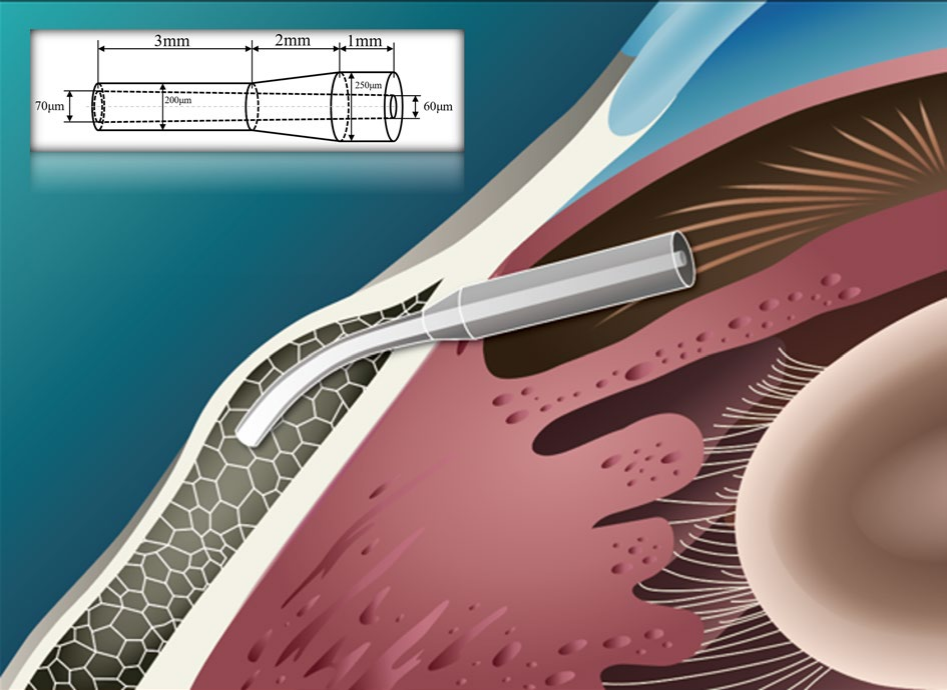
Schematic illustration of drainage stent implantation with deformation due to tissue compression (upper left corner shows a schematic representation of the stent size)

Figure 8 shows the stent implantation. The total length of the stent is 6 mm and the outer diameter is distributed in a stepped pattern: the outer diameter varies along the shaft: 250 μm over 1 mm, reducing to 200 μm over 3 mm, bridged by a 2 mm transitional segment. The stent features a lumen with a cross-sectional expansion: at one end of the outer diameter 250 μm, the inner diameter is 60 μm, and at the other end of the outer diameter 200 μm, the inner diameter is 70 μm. After the micro-drainage stent is implanted into the eye, the caudal end of the stent should be maintained at about 1–2 mm in the subconjunctival space, about 3 mm in the scleral tissue, and about 1 mm in the anterior chamber to prevent the trabecular tissue from blocking the drainage outlet. After implantation, the stent is compressed and deformed by the tissue due to the curvature of the cornea. This concentration of force typically occurs in the middle of the drainage stent [13, 25]. Previous clinical implantation of commercial drainage stents has shown the occurrence of intermediate fractures. Excessive stress concentration is easily identified as the cause of the fractures.

Upon implantation of the drainage stent into the eye, the front half remains straight while protruding into the tissue, whereas the second half bends and deforms in response to the curvature of the sclera, resulting in stress concentration during the middle bending deformation. A finite element analysis of the stent forces was performed utilizing the Solid Mechanics module in COMSOL 5.6. The boundary condition for the flat part of the first half is set as fixed support, assuming no displacement occurs following the implantation of the drainage stent. Furthermore, the second half of the stent experiences a radial force, which is treated as a uniform load due to the small size of the stent. The drainage stent had a material density of 2 kg/m^3^, a Poisson’s ratio of 0.4, and a Young’s modulus of 204 MPa. We use tensile machines (HY-0350, HENG WING PRECISION INSTRUMENT Co., LTD) to characterize the mechanical parameters of this material. By weighing the mass of a single tensile specimen, then dividing by the volume of the standardized tensile specimen model, we can derive the density parameter of the material. The stress concentration distribution of the diverging drainage stent in this paper and the stress concentration distribution of the commercial glaucoma drainage stent XEN after implantation were analyzed by simulation, and the stress concentration value of the bending part was compared, and the bending resistance of the stent was analyzed.

### Materials and fabrication

Previous clinical implantation of commercial drainage stents has shown the occurrence of intermediate fractures [13, 25]. Excessive stress concentration is easily identified as the cause of the fractures. Therefore, the stent must have a certain strength to be able to exist stably in the eye for a long time and avoid being broken. The soft texture can make the stent adapts to the tissue shape, becoming tissue conforming. According to our group's previous research, the biocompatible materials mixed by SR348OP (ARKEMA Sartomer), Bis-GMA (ARKEMA Sartomer) and PEG600MA (ARKEMA Sartomer) in accordance with the ratio of 7:3:5 were finally selected. This ratio can take into account the strength and flexibility of the stent at the same time. The selection of material ratio comes from the previous research of our group [26].

**Fig. 9:**
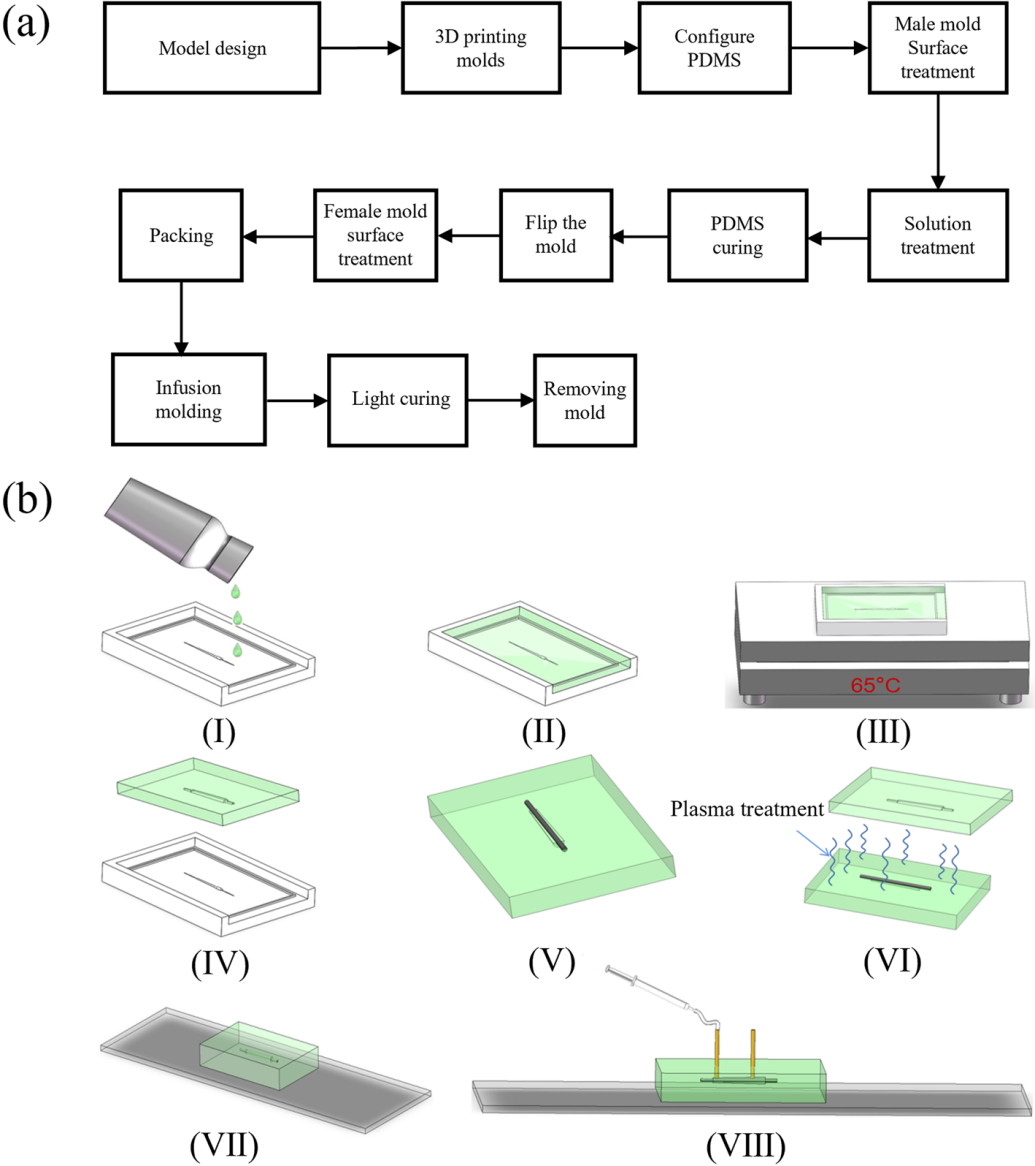
**a** Flowchart of drainage stent preparation; **b** fabrication process of a microfluidic device incorporating a drainage stent. **I** Pouring PDMS onto a master mold treated with surface modifications. **II** Filling the mold with a PDMS solution. **III** Curing the PDMS on a thermostatic heating table at 65 °C. **IV** Removing the PDMS chip from the mold after curing. **V** Placing a tungsten steel needle in the guiding groove of the mold. **VI** Subjecting the chip to plasma treatment with the inserted tungsten steel needle and a matching chip. **VII** Packaging the chip **VIII** Injecting stent material into the chip using a syringe pump

The fabrication process of drainage stents is mainly divided into two stages, namely the microfluidic chip preparation stage and the packaging molding stage. The process flowchart is presented as Fig. 9a.

First, the mold is designed and printed using a DLP printer. We used light-curing 3D printing technology to print the mold for subsequent mold turning, which is a very common method in the field of microfluidic chip preparation. The DLP 3D printer (Shape 1 +, RAYSHAPE 3D Co., Suzhou, China) we use can have a minimum layer height of 25 μm, and the final mold size is 3 cm*5 cm*0.8 cm. The PDMS solution (Sylgard 184, Dow Corning) and PDMS curing agent (Sylgard 184, Dow Corning) are mixed thoroughly in a ratio of 10:1, and any bubbles generated during the stirring and mixing process are removed using a vacuum pump. The solution is then poured into the mold, and another round of vacuuming is performed to remove any air trapped the surface of the mold the pouring process. Next, the mold is placed on a thermostatic heating table, and the temperature is adjusted to 55–75 °C. The mold is heated for a period of 2–6 h to facilitate the curing of the PDMS. Finally, the PDMS curing chip is carefully removed from the mold and covered with a preservative film to protect it.

**Fig. 10:**
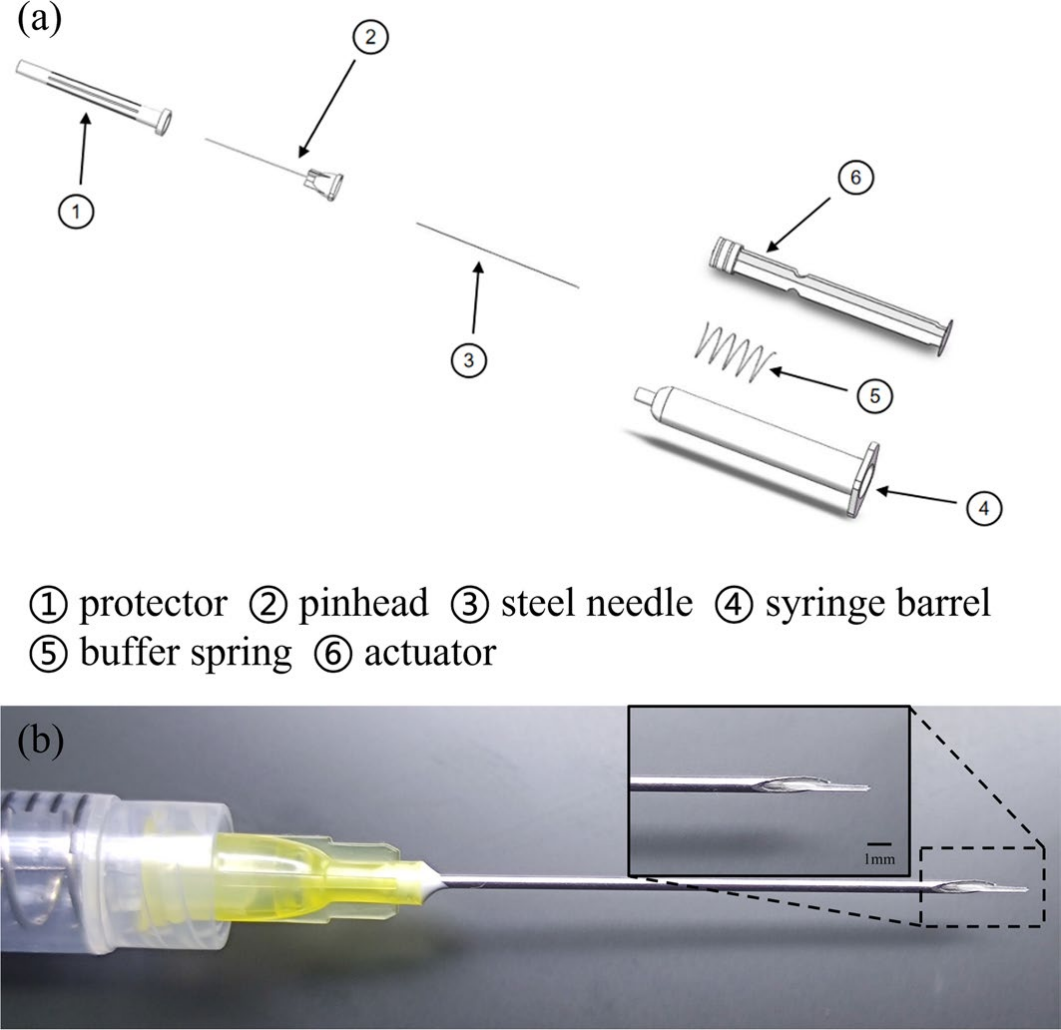
**a** Explosion diagram of the drainage stent injection system; **b** fit of the injection system with the stent

The process of chip packaging and infusion molding consists of 8–12 steps, as shown in the flowchart (Fig. 9b). Initially, the mold is placed into a plasma cleaning machine (PDC-MG, CHENGDU MINGHENG S&T CO., LTD) and subjected to oxygen treatment for approximately 10 min (30W, initial vacuum level 1.0E5). This step is to allow the PDMS mold to be bonded (Fig. 10b–f). PDMS has good hydrophobicity after curing, and in order for the liquid to pass through the microchannel smoothly and make the molded stent structure uniform and complete, it must be changed from hydrophobic to hydrophilic. Microfluidic chips fabricated using PDMS can be bonded to a variety of substrate materials through plasma-modified bonding processes, which are widely used in the field of microfluidics. Then, the perfusion port and drain port are carefully chosen before proceeding with the packaging process. At the same time, lumen construction metal (a tungsten steel needle of non-cylindrical outer diameter, which has a certain degree of flexibility and is not easily breakable) is required for coaxial placement. This step is to remove the needle to form a non-cylindrical lumen after the stent has been cured. After placing the tungsten steel needle and bonding the upper and lower halves of the chip, the stent material is injected from the injection port with a needle until the chip cavity is filled. Then, it is cured by exposure to a 365 nm ultraviolet curing lamp for approximately 20 s. Subsequently, the drainage stent is removed from the mold and soaked in alcohol (75%, General reagent). This is to facilitate the subsequent removal of the cured stent from the mold. After a specific duration, the stent is retrieved and the tungsten steel needle is removed. Following that, the samples are soaked in cysteine (Sigma-Aldrich), undergo surface treatment. This is an amino acid that contains sulfhydryl functional groups. The sulfhydryl functional group of the amino acid undergoes an addition reaction with the unreacted acrylic ester double bonds on the drainage stent, preventing residual double bonds from causing cytotoxicity and thus enhancing the surface biocompatibility of the drainage stent. It is then sterilized, sealed, and finally dried for preservation.

### Injection setup

The minimally invasive injection implant system plays a crucial role in MIGS devices used for glaucoma treatment. The injection system needs to fulfill several functions, including puncturing, delivering the medicine, and providing support to the implant, to meet the clinical requirements of minimally invasive implantation. For the details required for the exercise of each function, specific design requirements are made, such as the encapsulated shell, one is to support the internal pusher, guide the pusher to propulsion, and also pay attention to the shell to facilitate the surgeon to hold the bolus. The puncture function should pay attention to the diameter of the puncture needle should not be too large. The bolus function needs to pay attention to the smoothness and smoothness of the bolus. Because the inside of the eye is a confined space, air is expelled into the anterior chamber of the eye during the syringe, so the pressure balance also needs to be considered.

Before assembly, the parts of the injection system are sterilized, the metal parts of the injection system are sterilized at high temperature, and the plastic parts are irradiated. The puncture needle of the injection system should be matched with the outer diameter of the drainage stent. The stent’s maximum outer diameter is 250 μm, for which we opt for 20–22G needles to ensure compatibility. Given that the stent employs a hydrogel material capable of water absorption and swelling, the needle’s inner diameter should slightly exceed the maximum outer diameter of the stent, allowing ample room for swelling. By adding a buffer spring to the syringe barrel, it is convenient for ophthalmologists to perform surgery with stable bolus injection and easy force control. A built-in steel needle is designed at the top of the actuator, and the steel needle fits with the gap between the needles outside the injection implantation system to achieve the stent bolus function. The assembly process of the injection system involves several steps (Fig. 10a). Firstly, the inner steel needle is inserted into the actuator's holster. Then, a spring is placed on the steel needle. Next, an ordinary needle is used to connect the inner steel needle in the syringe barrel, guiding it out of the syringe. The steel needle is then threaded into the external pinhead, and finally, the pinhead is screwed into the syringe. Once these steps are completed, the injection system is fully assembled. Finally, it should be stored in a light-protected sterile environment. Figure 10b illustrates the fit of the injection system with the stent.

### Degradation properties of the stents in vitro

The drainage stent is implanted in the eye for an extended period of time, and besides its drainage function, degradation stability is of paramount importance. Considering the small mass of the individual stent and the accuracy of the instrument, the enlarged samples are made for degradation experiments with the mass of about 1.3 mg per stent. The 10 stents are considered as one parallel sample, for a total of 5 parallel samples. The measurement time is 1, 2, 3, 6, 9, and 12 months from the start date. The specific steps for degradation experiments are as follows: weighing of a parallel sample using a balance, recorded as W_0_. The specimen holder was subsequently placed in a 5-ml centrifuge tube, followed by the addition of 4 ml of PBS (pH = 7.4) (HyClone). The parallel samples were then placed in a constant temperature shaking box at 37 °C and 80 rpm. The stents were immersed in PBS for 1, 2, 3, 6, 9, and 12 months, respectively. At each measurement time point, the specimen is removed, and the weight of each parallel sample is measured after drying, recorded as Wfd. Each set of data is the average of five parallel samples. The degradation rate is calculated as follows:

10 $$\text{Degradation rate}\left(\text{\%}\right)=\frac{{W}_{0}-{W}_{f\,d}}{{W}_{0}}.$$

As previously mentioned, in the manufacturing process of drainage stents, cysteine soaking surface treatment is employed, along with the introduction of photoinitiators. Therefore, the precipitates that may be present in the drainage stent manufacturing process include amino acids, additive material molecules, residual monomers, and so on. In order to further determine the residue of harmful substances in the treated stent, the stent extract was extracted for infrared spectroscopy measurement (cm 6700, Thermo Nicolet), and the precipitate of the stent was analyzed by gas chromatography–spectrometry (AB Sciex 4800 plus). Infrared spectroscopy can provide information about compounds in a precipitated solution, and by comparing their spectra with known standards, it is possible to determine the compounds, functional groups, etc., that may be present in the solution, provide detailed information about the chemical changes during the degradation process of materials, and help to understand the degradation mechanism and product characteristics of materials. Mass spectrometry has a similar effect to infrared spectroscopy. Mass spectrometry can determine the molecular structure of degradation products, including their chemical formula and carbon backbone. After obtaining the mass spectrum of the stent precipitation solution, determine if there is any hazardous material residue in the scaffold by comparing the ion peaks on the sample mass spectrum to those on the 2959 initiator and cysteine standard mass spectra. Each centrifuge tube is equipped with a drainage stent, and a total of six groups are set up, with each group containing three samples (one to be tested and two backups). Add 4 ml of PBS (pH = 7.4) to each centrifuge tube, place them in a constant temperature shaking chamber (37 °C, 1000 rmp), and take the immersion solution for testing after 3 days.

### Biocompatibility

#### Cytotoxicity assessment of the stents

HUVEC cells were cultured in leach liquor and subjected to three days of live/dead staining. Calcein-AM and PI stains (C326/P378, DOJINDO LABORATORIES) were employed for discriminating between viable and non-viable cells. Due to its lipophilicity, Calcein-AM can penetrate the cell membrane of viable cells. It is then cleaved by intracellular enzymes, resulting in the formation of Calcein, which becomes trapped within the cell membrane and emits green fluorescence at a wavelength of 490 nm. PI stains can permeate the membranes of non-viable cells, enter the nucleus, and intercalate with nuclear DNA, leading to the detection of red fluorescence at 488 nm or 545 nm [24]. HUVEC cells were seeded at a density of 3 × 10^3^ cells per well in 48-well plates following the standard cell culture protocol. The experimental group was treated with the medium containing the leach liquor, while the blank control group was treated with plain Dulbecco’s modified Eagle medium (DMEM (HyClone)) for comparison. Live/dead staining of cells was performed on days 1, 2, and 3 to determine fluorescence.

The specific steps are as follows:

(I.) Prepare the cell live/dead staining reagent by weighing 1 mg of PI and Calcein-AM. Dissolve them separately in 1 ml of deionized water and 1 ml of dimethyl sulfoxide [DMSO (Sigma-D2650)] to obtain a 1 mM Calcein-AM solution and a 1.5 mM PI solution. Store the solutions in a dark refrigerator at − 20 °C. (II.) Mix 4 μl of Calcein-AM solution and 6 μl of PI solution, and dissolve them in 2 ml of PBS solution to create the cell live/dead staining solvent. (III.) Using a pipette, aspirate the cell culture medium to be tested, wash the dish with PBS solution 3–5 times, add 200 μl of staining solvent per well, and incubate the dish for 30 min. (IV.) Remove the staining solvent from the medium, and capture images using an inverted fluorescence microscope in a light-free environment. Three random fields of view were examined per well.

#### Subcutaneous implantation experiments of SD rats

A total of twenty-one 6-week-old Sprague–Dawley (SD) rats were divided into four groups: three experimental groups (each with five rats) and one control group (with six rats). The SD rats were given an intramuscular injection of 0.4–0.6 ml (5 mg/kg) of xylazine hydrochloride and an injection of pentobarbital sodium (30 mg/kg) into the vein at the ear margin to induce combined anesthesia. The back hair of the rats was shaved, and a 2-cm incision was made using a scalpel. In the experimental group, drainage stents were implanted under the skin, while the control group did not receive any implantation after surgery. Tissue sections were observed at two weeks, four weeks, and three months post-surgery. The sections were stained with hematoxylin and eosin (HE), and the tissue inflammatory response was compared between the experimental group and the control group.

### Animal surgery and stent implantation

Adult New Zealand white (NZW) rabbits were used as experimental animals and acclimated to the animal room environment for one week before the experiment [27]. The ambient temperature was controlled with a 12-h light/12-h dark cycle. During the acclimation period, the rabbits’ ears or other body parts were marked with markers or dyes, and they were divided into groups. This experiment was approved by the Laboratory Animal Ethics Committee of Zhongshan Ophthalmology Center, Sun Yat-sen University. To evaluate the system in vivo, drainage stents were implanted in the right eye of normal NZW rabbits with high IOP. Daily recordings of changes in intraocular pressure, follicular size, and vascular density grade were conducted to assess the functional and biological safety of the implanted drainage stents. Follow-up measurement of intraocular pressure and observation of follicle size and vascular density after surgery can provide an assessment of the outcome of the procedure and the condition of the eye after surgery. The filtration vesicle is a new aqueous humor drainage channel formed after stent implantation, and its size and shape directly affect the efficiency of aqueous humor drainage. Larger vesicles usually mean better aqueous humor drainage, which helps to reduce intraocular pressure. Vascular density reflects the blood supply to the tissues surrounding the vesicles, and higher vessel density usually means better tissue blood supply, contributing to the normal function and stability of the vesicles. According to the glaucoma filter vesicle IBAGS classification, the vascular density grade is divided into 0–4 grades. The higher the grade, the greater the vascular density. The size of the follicle is the diameter of the follicle (mm) * the height of the vertical cornea (mm) * the height from the sclera (mm).

**Fig. 11:**
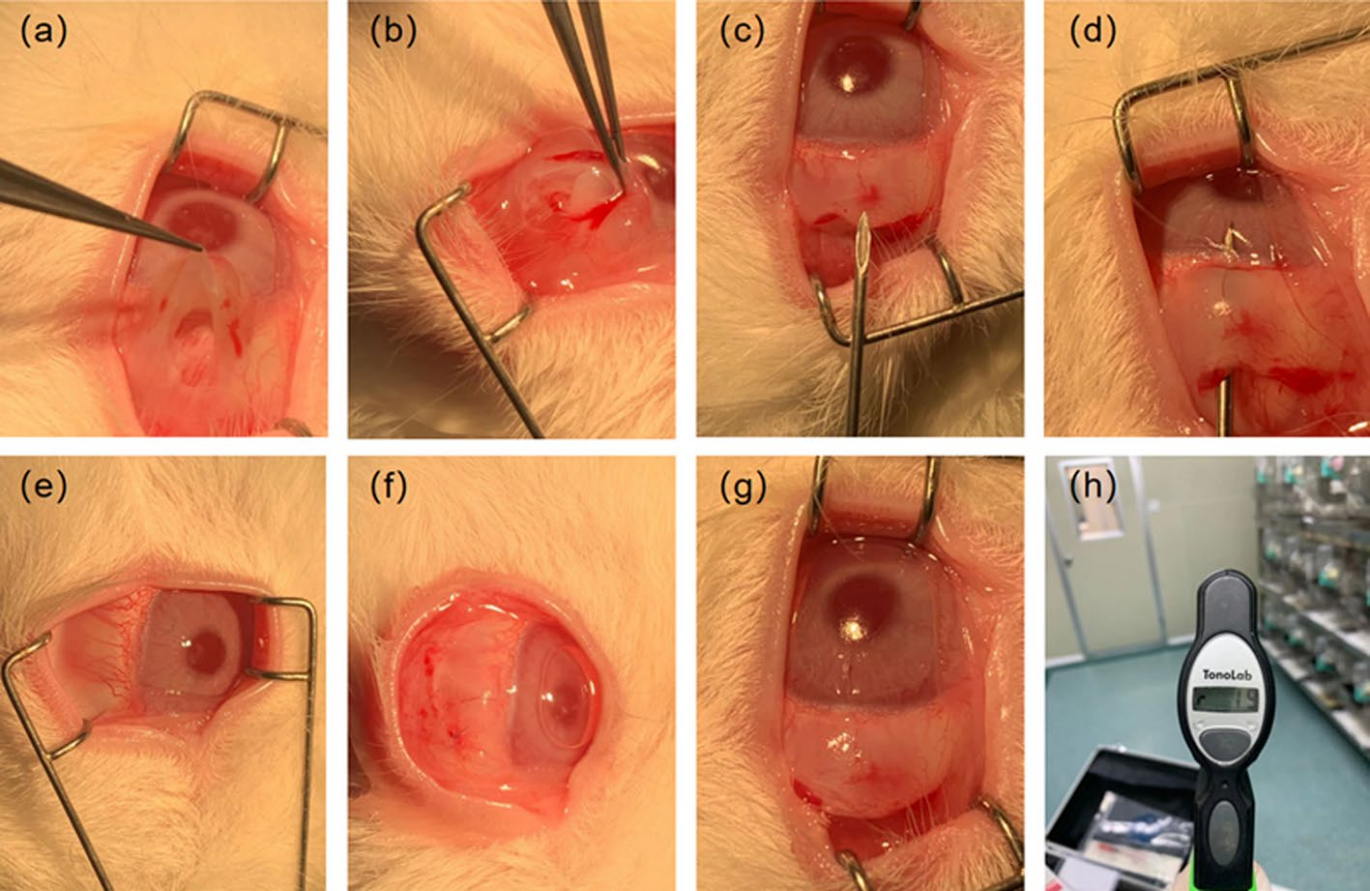
**a–g** The procedure of implanting the drainage stent in the rabbit eye; **h** the measurement of intraocular pressure in the rabbit eye using the iCare Handheld Tonometer one day post-implantation

NZW rabbits were randomly divided into four groups (T1–T4), with one rabbit in each group, and housed individually. Groups T1–T3 underwent drainage stent implantation, while Group T4 served as the control group. The surgical procedure is shown in Fig. 11a–h (I.) The rabbits received intramuscular injections of 0.4–0.6 ml (5 mg/kg) of Xylazine Hydrochloride and pentobarbital sodium (30 mg/kg) was injected into the vein at the ear margin to induce combined anesthesia. Ocular local anesthesia was achieved using oxybuprocaine hydrochloride eye drops (s.a. ALCON-COUVREUR n.v.). (II.) The NZW rabbit was positioned on its left side to expose the right eye. The eyelashes of the New Zealand white rabbit were trimmed, and the area around the eyelids was disinfected with Aner iodine (Likang High-tech). The conjunctival sac was then soaked in povidone iodine for 1 min and rinsed multiple times with stroke-physiological saline solution (SPSS). (III.) Prior to surgery, the eyelid was opened using an eye speculum to expose the upper surgical conjunctival area. Local infiltration anesthesia was administered using lidocaine. A conjunctival flap was created along the limbus, and the conjunctiva and subconjunctival tissue were dissected bluntly along the scleral plane until reaching the limbus. (IV.) A needle was used to create a corneal scleral tunnel 2 mm behind the corneoscleral limbus. Subsequently, a drainage stent was implanted along the tunnel, and the conjunctival injection incision was continuously sutured using nylon thread. After the procedure, erythromycin eye ointment (Cisen pharma) was applied to the incision to prevent infection. The eye speculum was removed, and the rabbit was assisted in closing its eyelids. Once it recovered, it was returned to its cage.

The surgical procedure was performed by the same experienced surgeon. Postoperative care involved administering daily drops of Tobramycin and Dexamethasone Eye Drops. Daily recordings of changes in intraocular pressure, follicular size, and vascular density grade were conducted to assess the functional and biological safety of the implanted drainage stents. Follow-up measurement of intraocular pressure and observation of follicle size and vascular density after surgery can provide an assessment of the outcome of the procedure and the condition of the eye after surgery. The filtration vesicle is a new aqueous humor drainage channel formed after stent implantation, and its size and shape directly affect the efficiency of aqueous humor drainage. Larger vesicles usually mean better aqueous humor drainage, which helps to reduce intraocular pressure. Vascular density reflects the blood supply to the tissues surrounding the vesicles, and higher vessel density usually means better tissue blood supply, contributing to the normal function and stability of the vesicles. According to the glaucoma filter vesicle IBAGS classification, the vascular density grade is divided into 0–4 grades. The higher the grade, the greater the vascular density. The size of the follicle is the diameter of the follicle (mm) * the height of the vertical cornea (mm) * the height from the sclera (mm).

### Statistical analyses

Statistical analyses were performed using SPSSAU. The data were presented as the mean ± standard deviation (SD) and were analyzed using RM ANOVA and one-way ANOVA. A *p* value < 0.05 was used as a criterion for statistical significance.

## Data Availability

The datasets used and/or analyzed during the current study are available from the corresponding author on reasonable request.

## Abbreviations

IOP: Intraocular pressure
MIGS: Minimally invasive glaucoma surgery
HUVEC: Human umbilical vein endothelial cells
SD rats: Sprague–Dawley rats
NZW rabbits: New Zealand white rabbits
DLP: Digital light processing
PDMS: Polydimethylsiloxane
PBS: Phosphate buffered saline
DMEM: Dulbecco’s modified Eagle medium
HE: Hematoxylin and eosin
SPSS: Stroke-physiological saline solution

## References

[1] Tham YC, Li X, Wong TY, et al. Global prevalence of glaucoma and projections of glaucoma burden through 2040: a systematic review and meta-analysis. Ophthalmology. 2014;121(11):2081–90. 24974815 10.1016/j.ophtha.2014.05.013

[2] Bugara K, Pacwa A, Smedowski A. Molecular pathways in experimental glaucoma models. Front Neurosci. 2024;18:1363170. 38562304 10.3389/fnins.2024.1363170 PMC10982327

[3] Li M, Gao ZL, Zhang QP, et al. Autophagy in glaucoma pathogenesis: therapeutic potential and future perspectives. Front Cell Dev Biol. 2022;10:1068213. 36589756 10.3389/fcell.2022.1068213 PMC9795220

[4] Nickells RW, Howell GR, Soto I, et al. Under pressure: cellular and molecular responses during glaucoma, a common neurodegeneration with axonopathy. Annu Rev Neurosci. 2012;35:153–79. 22524788 10.1146/annurev.neuro.051508.135728

[5] Saheb H, Ahmed IIK. Micro-invasive glaucoma surgery: current perspectives and future directions. Curr Opin Ophthalmol. 2012;23(2):96–104. 22249233 10.1097/ICU.0b013e32834ff1e7

[6] Ang BCH, Lim SY, Betzler BK, Wong HJ, Stewart MW, Dorairaj S. Recent advancements in glaucoma surgery—a review. Bioengineering (Basel). 2023;10(9):1096. 37760198 10.3390/bioengineering10091096 PMC10525614

[7] Richter GM, Coleman AL. Minimally invasive glaucoma surgery: current status and future prospects. Clin Ophthalmol. 2016:189–206. 10.2147/OPTH.S80490 PMC4734795 26869753

[8] Schultz T, Schojai M, Kersten-Gomez I, et al. Ab externo device for the treatment of glaucoma: direct flow from the anterior chamber to the ocular surface. J Cataract Refract Surg. 2020;46(7):941–3. 32271273 10.1097/j.jcrs.0000000000000202

[9] Feuer WJ, Budenz, et al. Postoperative complications in the tube versus trabeculectomy (TVT) study during five years of follow-up. 10.1016/j.ajo.2011.10.024 PMC3653167 22244522

[10] King AJ, Hudson J, Fernie G, et al. Primary trabeculectomy for advanced glaucoma: pragmatic multicentre randomised controlled trial (TAGS). BMJ. 2021;373:n1014. 33980505 10.1136/bmj.n1014 PMC8114777

[11] Lavia C, Dallorto L, Maule M, et al. Minimally-invasive glaucoma surgeries (MIGS) for open-angle glaucoma: a systematic review and meta-analysis. PLoS ONE. 2017;12(8): e0183142. 28850575 10.1371/journal.pone.0183142 PMC5574616

[12] Batlle JF, Fantes F, Riss I, et al. Three-year follow-up of a novel aqueous humor microshunt. J Glaucoma. 2016;25(2):58–65. 10.1097/IJG.0000000000000368 26766400

[13] Lewis RA. Ab interno approach to the subconjunctival space using a collagen glaucoma stent. J Cataract Refract Surg. 2014;40(8):1301–6. 24943904 10.1016/j.jcrs.2014.01.032

[14] Kerr NM, Ahmed IIK, Pinchuk L, et al. PRESERFLO MicroShunt. Minimally Invasive Glaucoma Surgery. 2021:91–103.

[15] Pinchuk L, Riss I, Batlle JF, et al. The development of a micro-shunt made from poly (styrene-block-isobutylene-block-styrene) to treat glaucoma. J Biomed Mater Res B Appl Biomater. 2017;105(1):211–21. 26380916 10.1002/jbm.b.33525 PMC5215625

[16] Pfeiffer N, Garcia-Feijoo J, Martinez-De-la-casa JM, et al. A randomized trial of a Schlemm’s canal microstent with phacoemulsification for reducing intraocular pressure in open-angle glaucoma. Ophthalmology. 2015;122(7):1283–93. 25972254 10.1016/j.ophtha.2015.03.031

[17] Saptaji K, Gebremariam MA, Azhari MABM. Machining of biocompatible materials: a review. Int J Adv Manuf Technol. 2018;97:2255–92.

[18] Du X-Y, Li Q, Wu G, et al. Multifunctional micro/nanoscale fibers based on microfluidic spinning technology. Adv Mater. 2019;31(52):1903733. 10.1002/adma.201903733 31573714

[19] Gedde SJ, Herndon LW, Brandt JD, et al. Postoperative complications in the Tube Versus Trabeculectomy (TVT) study during five years of follow-up. Am J Ophthalmol. 2012;153(5):804–14. 22244522 10.1016/j.ajo.2011.10.024 PMC3653167

[20] Karlova EV, Radaykina MV. Valved and non-valved drainage systems in the surgical treatment of refractory glaucoma. Ophthalmol Russia. 2019;16:123–6.

[21] Kouhyar T, Bozena K, Maleki TJ. Novel micromachined valved glaucoma drainage devices. In: 2006 International Conference of the IEEE Engineering in Medicine and Biology Society. 2006; pp. 6364–9. 10.1109/IEMBS.2006.260421. 10.1109/IEMBS.2006.260421 17947189

[22] Rosentreter A, Schild AM, Dinslage S, et al. Biodegradable implant for tissue repair after glaucoma drainage device surgery. J Glaucoma. 2011;21(2):76–8. 10.1097/IJG.0b013e3182027ab0 21278584

[23] Jiang X, Zhou Z, Huang X, et al. Micronozzle/diffuser flow and its application in micro valveless pumps. Sens Actuators, A. 1998;70(1–2):81–7.

[24] Jang YY, Cho D, Kim SK, et al. An improved flow cytometry-based natural killer cytotoxicity assay involving calcein AM staining of effector cells. Ann Clin Lab Sci. 2012;42(1):42–9. 22371909

[25] Chaudhary A, Salinas L, Guidotti JM, Mermoud A, Mansouri K. XEN Gel Implant: a new surgical approach in glaucoma. Expert Rev Med Devices. 2018;15:47–59. 29258404 10.1080/17434440.2018.1419060

[26] Men L, Wang K, Hu N, et al. Biocompatible polymers with tunable mechanical properties and conductive functionality on two-photon 3D printing. RSC Adv. 2023;13(13):8586–93. 36926305 10.1039/d2ra07464h PMC10013736

[27] Acosta AC, Espana EM, Yamamoto H, et al. A newly designed glaucoma drainage implant made of poly(styrene-b-isobutylene-b-styrene): biocompatibility and function in normal rabbit eyes. Arch Ophthalmol. 2006;124(12):1742–9. 10.1001/archopht.124.12.1742. 17159034 10.1001/archopht.124.12.1742

[28] Verticchio Vercellin AC, Harris A, Oddone F, et al. Ocular blood flow biomarkers may predict long-term glaucoma progression. Br J Ophthalmol. 2023;108:946. 10.1136/bjo-2022-322644 PMC12333459 37852742

[29] Hollo G. Progressive decrease of peripapillary angioflow vessel density during structural and visual field progression in early primary open-angle glaucoma. J Glaucoma. 2017;26(7):661–4. 28557829 10.1097/IJG.0000000000000695

[30] Ruiz-Baier R, Taffetani M, Westermeyer HD, Yotov I. The Biot-Stokes coupling using total pressure: formulation, analysis and application to interfacial flow in the eye. Comput Methods Appl Mech Eng. 2022;389:114384.

