# Correction: A glaucoma micro-stent with diverging channel and stepped shaft structure based on microfluidic template processing technology

**DOI:** 10.1186/s12938-025-01358-9

**Published:** 2025-03-06

**Authors:** Chen Wang, Fule Wang, Yunru Liao, Chengguo Zuo, Mingkai Lin, Kemin Wang, Dongni Ren, Hongbo Zhang, Ruixue Yin

**Affiliations:** 1 School of Mechanical and Power Engineering, East China University of Science and Technology, No. 130 Meilong Road, Shanghai, 200237 China; 2 State Key Laboratory of Ophthalmology, Zhongshan Ophthalmic Center, Sun Yat-Sen University, No.7 Jinsui Road, Tianhe District, Guangzhou, 510060 China; 3 Department of Ophthalmology, Sun Yat-Sen Memorial Hospital, Sun Yat-Sen University, Guangzhou, China; 4 Mingche Biotechnology Co., Ltd, Suzhou, 215000 China

**Correction: BioMedical Engineering OnLine (2024) 23:73** 10.1186/s12938-024-01266-4

In this article [1], Fig. 4 appeared incorrectly and have now been corrected in the original publication. For completeness and transparency, both correct and incorrect versions are displayed below.

The original article has been corrected.

Incorrect Fig. 4
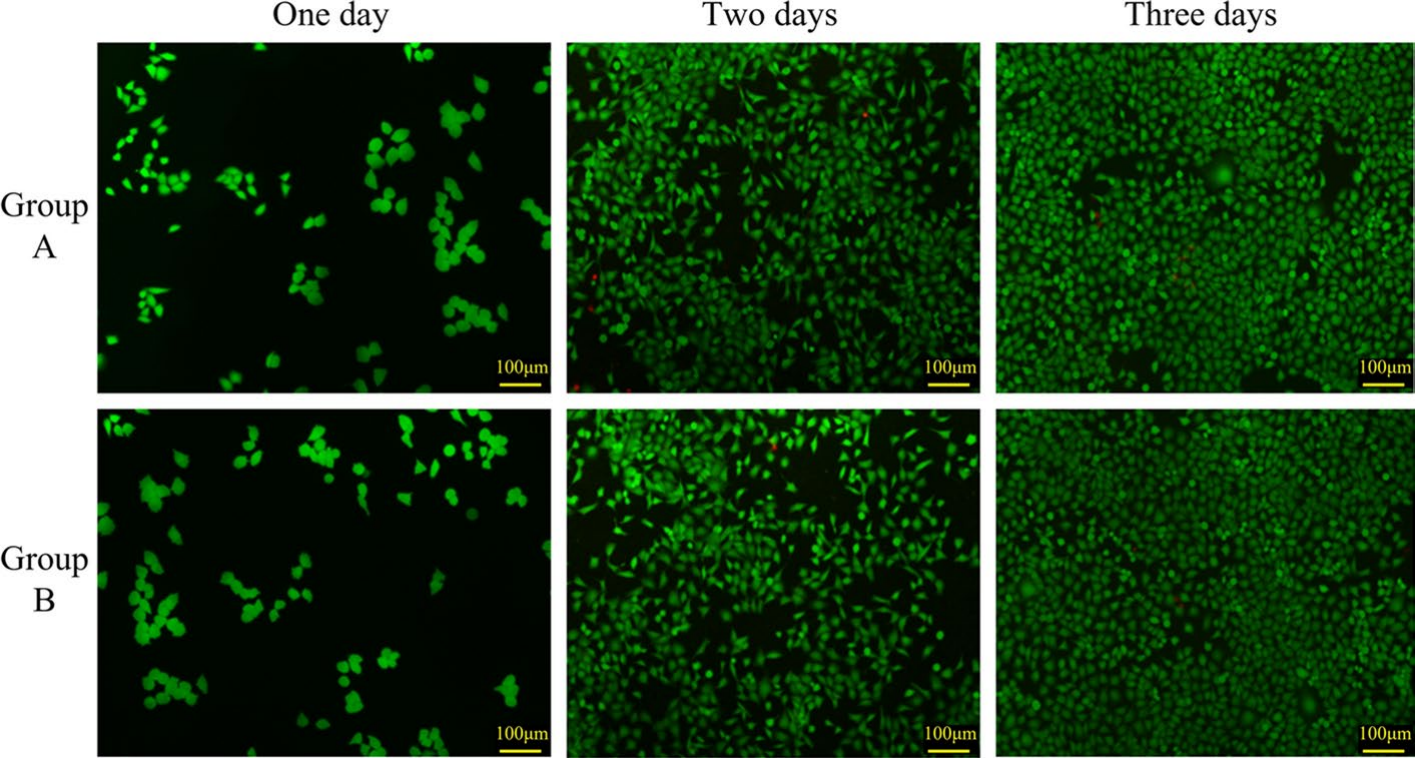


**Fig. 4** HUVEC cell culture subjected to dead and alive staining. Group A is the blank control group, and Group B is the experimental group treated with the extraction

Correct Fig. 4

**Fig. 4:**
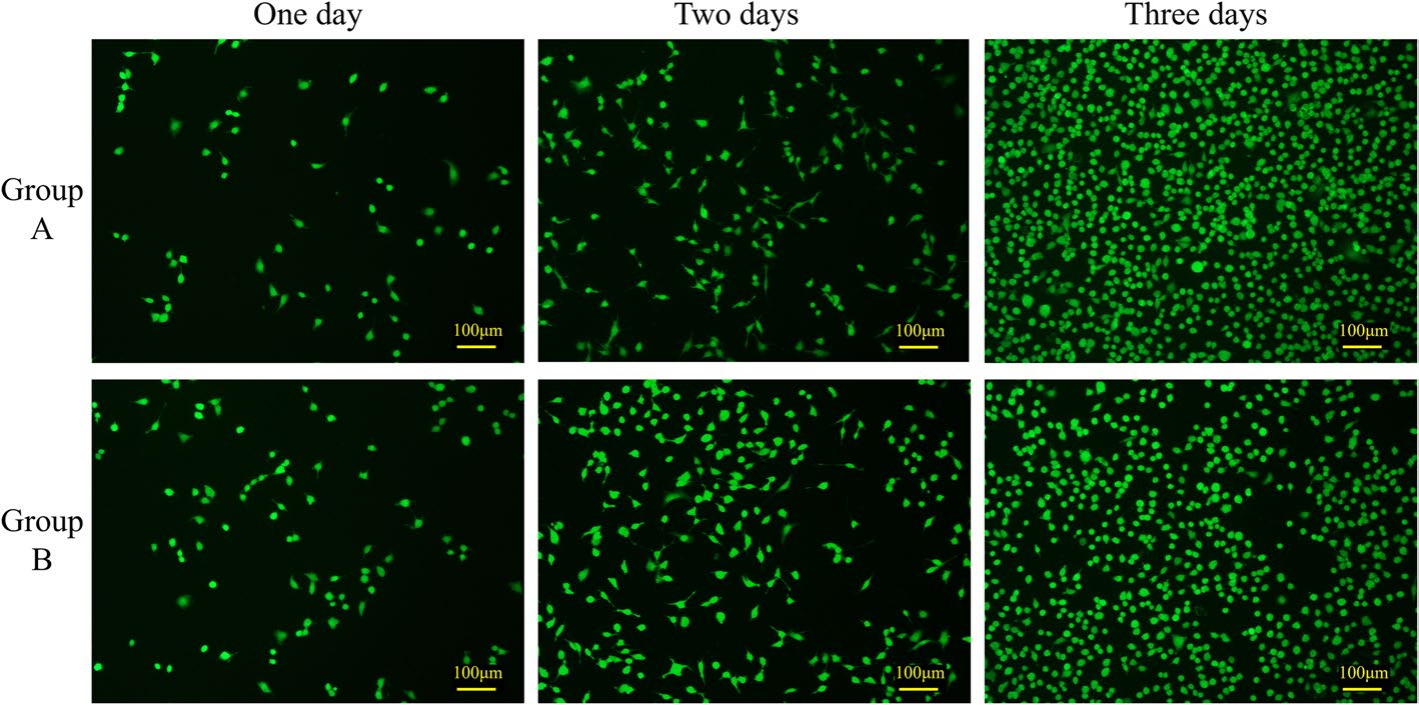
HUVEC cell culture subjected to dead and alive staining. Group A is the blank control group, and Group B is the experimental group treated with the extraction
